# Checklist of ladybirds of Algeria with two new recorded species (Coleoptera, Coccinellidae)

**DOI:** 10.3897/zookeys.774.23895

**Published:** 2018-07-12

**Authors:** Mohamed Amin Lakhal, Djelloul Ghezali, Oldřich Nedvěd, Salaheddine Doumandji

**Affiliations:** 1 Department of Agricultural and Forest Zoology, Agronomic National School Superior of El Harrach, Algeria Avenue Hassan Badi, 16200, El Harrach, Algeria; 2 Faculty of Science, University of South Bohemia, Branišovská 1760, 370 05 Ceské Budejovice, Czech Republic; 3 Institute of Entomology, Biology Centre AS CR, Branišovská 31a, 370 05 Ceské Budejovice, Czech Republic

**Keywords:** Africa, aphids, *Coccinelloidea*, distribution, ladybug, taxonomy

## Abstract

An updated and corrected checklist of species of ladybird beetles (Coleoptera: Coccinellidae) known in Algeria now contains 75 species belonging in ten tribes. New country records include the European species *Oenopia conglobata* and the invasive Asian species *Harmonia axyridis*. Sampling data is provided for 14 species found during a faunistic survey performed mostly in agroecosystems, together with host plant and prey species.

## Introduction

Family Coccinellidae (ladybirds) is the most species-rich family in the recently recognized beetle superfamily Coccinelloidea (Robertson et al. 2015) with approximately 6000 species described worldwide (Vandenberg 2002). They are mostly beneficial insects, their larvae and adults feeding on pests, especially on scale insects and aphids (Hodek et al. 2012, Giorgi et al. 2009).

Among beetle families, ladybird beetles (Coccinellidae) of individual countries are relatively well known, and the fauna of Algeria is also relatively well documented (Saharaoui and Gourreau 2000, Kovář 2007, Saharaoui et al. 2014).During a recent relatively limited survey, we found two species recorded for the first time in Algeria which need to be added to the list. In preparing the checklist, we also found many taxonomical errors in the previous species lists or old taxonomy that was recently changed mainly due to molecular phylogenetic studies. Thus, we provide an updated and corrected checklist of species of the family Coccinellidae in Algeria.

During the faunistic survey performed mostly in agroecosystems, we found 12 species reported before and therefore we provide details of their localities and dates of sampling as well as their host plants and prey species.

## Materials and methods

Literature records were reviewed to set up up-to-date list of species of Coccinellidae recorded from Algeria. Our sampling was performed to confirm species occurrence and their host/food relationships as a part of study on the natural enemies of aphids. Survey has been carried out in agroecosystems in distant localities within the country – name of locality, geographic coordinates, date of sampling and host plant are given in Table 1. Beetles were sampled from plants using sweeping net (the most effective method for Coccinellidae found by Kherbouche et al. 2015) and the Japanese umbrella. We also sampled plant fragments infested with aphids for their identification. Besides adults, also larvae of the ladybirds were collected for identification. Samples were preserved in 70% ethanol, adult beetles were subsequently allowed to dry. Insects were photographed by digital camera Lumenera Infinity 2 mounted on stereomicroscope Nikon SMZ 1500, operated by QuickPHOTO CAMERA software. Series of images was stacked using Zerene Stacker 1.04. Species were identified using various available keys, such as Iablokoff-Khnzorian (1982), Nedvěd (2015). The specimens are deposited in Agronomic National School Superior of El Harrach, Algeria.

## Results

The updated checklist of Coccinellidae species of Algeria now includes the following 75 species assigned in ten tribes in the sense of Seago et al. (2011). Species taxonomy and synonymy follow Kovář (2007) and Nedvěd (2015). Species collected by the authors are marked with asterisk (*). The details of sampling regimes are listed in Table 1. Presence of herbivorous insects that may serve as food for the ladybirds is indicated in Table 2.

**Table 1.** Original records of the species of Coccinellidae in Algeria. Developmental stages, host plants or habitat, region of sampling, date of sampling, and coordinates are provided. The two species in bold are new records for Algeria.

Species	Adult	Larva	Plant/habitat	Region	Date	Coordinates
*Adalia bipunctata* (Linnaeus, 1758)	+	–	peach orchard	Mouzaia, BLIDA	10/04/2017	36°32'49"N, 2°41'47"E
*Adalia decempunctata* (Linnaeus, 1758)	3	0	*Ficus retusa*	El Harrach, ALGER	29/04/2017	36°43'02"N, 3°09'16"E
*Coccinella septempunctata* Linnaeus, 1758	+	–	pear orchard	Mouzaia, BLIDA	10 and 15/04/2017	36°32'51"N, 2°41'54‘‘E
	+	–	peach orchard	Mouzaia, BLIDA	10 and 15/04/2017	36°32'49"N, 2°41'47"E
	+	+	wheat field	Mouzaia, BLIDA	01/05/2017	36°32'55"N, 2°41'32"E
	+	–	alphalpha	(university) OUARGLA	04/04/2017	31°56'28"N, 5°18'20"E
	+	–	wheat field	ITDAS OUARGLA	02/04/2017	32°0'13"N, 5°27'58"E
	+	–	*Aristida* sp	Oued en Nsa, OUARGLA	04/04/2017	32°36'46"N, 4°57'43"E
	+	+	*Nerium oleander*	Mouzaia, BLIDA	10/05/2017	36°28'13"N, 2°41'29"E
	+	–	*Malva parviflora*	Mouzaia, BLIDA	08/05/2018	36°28'14"N, 2°41'29"E
	+	–	*Anthemis* sp.	Mouzaia, BLIDA	08/05/2018	36°28'14"N, 2°41'29"E
***Harmonia axyridis* (Pallas, 1773)**	**1**	**0**	**peach orchard**	**Mouzaia, BLIDA**	**10/04/2017**	**36°32'49"N, 2°41'46"E**
	**1**	**0**	–	**El Harrach, ALGER**	**05/12/2017**	**36°43'01"N, 3°09'16"E**
	**20**	**16**	***Malva parviflora***	**Mouzaia, BLIDA**	**08/05/2018**	**36°28'14"N, 2°41'29"E**
	**12**	**13**	***Notobasis syriaca***	**Mouzaia, BLIDA**	**08/05/2018**	**36°28'14"N, 2°41'29"E**
*Hippodamia variegata* (Goeze, 1777)	+	+	alphalpha field	(university) OUARGLA	04/04/2017	31°56'28"N, 5°18'20"E
	+	+	wheat field	ITDAS OUARGLA	03/04/2017	32°0'13"N, 5°27'58"E
	+	+	wheat field	Mouzaia, BLIDA	01/05/2017	36°32'54"N, 2°41'32"E
	+	–	wheat field	El Harrach, ALGER	17/04/2017	36°43'11"N, 3°09'03"E
	+	–	*Nerium oleander*	El Harrach, ALGER	05/05/2017	36°43'16"N, 3°9'5"E
	+	+	*Nerium oleander*	Mouzaia, BLIDA	10/05/2017	36°28'13"N, 2°41'29"E
	+	–	*Nerium oleander*	Mouzaia, BLIDA	08/05/2018	36°28'14"N, 2°41'29"E
	+	–	*Malva parviflora*	Mouzaia, BLIDA	08/05/2018	36°28'14"N, 2°41'29"E
	+	–	*Anthemis* sp.	Mouzaia, BLIDA	08/05/2018	36°28'14"N, 2°41'29"E
*Hyperaspis duvergeri* Fürsch, 1985	+	–	conifers	El Harrach, ALGER	09/03/2017	36°43'19"N, 3°08'58"E
*Hyperaspis marmottani* (Fairmaire, 1868)	+	–	peach orchard	Mouzaia, BLIDA	10/04/2017	36°32'49"N, 2°41'47"E
Nephus (Bipunctatus) peyerimhoffi (Sicard, 1923)	+	–	*Ficus retusa*	El Harrach, ALGER	29/04/2017	36°43'02"N, 3°09'16"E
***Oenopia conglobata* (Linnaeus, 1758)**	**1**	**0**	***Quercus ilex***	**El Harrach, ALGER**	**13/04/2017**	**36°43’14”N, 3°8’58"E**
	**1**	**0**	***Salpichroa origanifolia***	**El Harrach, ALGER**	**09/03/2017**	**36°43'13"N, 3°8'58"E**
	**1**	**0**	***Malva parviflora***	**Mouzaia, BLIDA**	**08/05/2018**	**36°28'13"N, 2°41'29"E**
	**1**	**0**	***Malva parviflora***	**Mouzaia, BLIDA**	**08/05/2018**	**36°28'14"N, 2°41'26"E**
	**1**	**0**	***Nerium oleander***	**Mouzaia, BLIDA**	**08/05/2018**	**36°28'14"N, 2°41'29"E**
*Oenopia doublieri* (Mulsant, 1846)	+	–	*Pittosporum tobira*	El Harrach, ALGER	16/03/2017	36°43'10"N, 3°09'00"E
*Psyllobora vigintiduopunctata* (Linnaeus, 1758)	+	–	*Salpichroa origanifolia*	El Harrach, ALGER	13/04/2017	36°43'14"N, 3°08'58"E
	+	–	*Pittosporum tobira*	El Harrach, ALGER	16/03/2017	36°43'10"N, 3°09'00"E
	+	–	*Citrus* sp.	Boufarik,Blida	18/03/2017	36°35'39"N, 2°55'8"E
*Rodolia cardinalis* (Mulsant, 1850)	+	+	*Pittosporum tobira*	El Harrach, ALGER	02/05/2017	36°43'05.2"N, 3°09'13"E
*Scymnus suffrianioides* Sahlberg, 1913	+	+	*Pittosporum tobira*	El Harrach, ALGER	17/04/2017	36°43'15"N, 3°8'59"E
*Stethorus pussilus* (Herbst, 1797)	+	–	*Pittosporum tobira*	El Harrach, ALGER	02/05/2017	36°43'05"N, 3°09'13"E

**Table 2.** Occurrence of aphid species on host plants that were visited by coccinellid predators.

Species	*Pyrus communis*	*Prunus persica*	*Triticum durum*	*Medicago sativa*	*Pittosporum tobira*	*Nerium oleander*	*Ficus retusa*	*Citrus sp.*	*Capsicum annuum*	*Malva parviflora*
*Acyrthosiphon pisum*				+						
*Aphididae* sp.							+			
*Aphis craccivora*				+						
*Aphis fabae*	+		+		+					
*Aphis gossypii*									+	
*Aphis nerii*						+				
*Aphis spiraecola*					+			+		
*Aphis umbrella*										+
*Dysaphis pyri*	+									
*Myzus persicae*	+	+							+	
*Rhopalosiphum padi*			+							

**Chilocorini**

*Chilocorus bipustulatus* (Linnaeus, 1758) (not *C. bipunctatus* as misspelled by Saharaoui‘ and Gourreau 2000; Kovář 2007; Saharaoui et al. 2014)

*Exochomus ericae* Crotch, 1874 (syn. *E. anchorifer* Bedel, 1885; syn. *Parexochomus anchorifer* (Allard, 1870) used by Saharaoui‘ and Gourreau 2000 and Saharaoui et al. 2014; Kovář 2007)

*Exochomus quadripustulatus* (Linnaeus, 1758) (source: Saharaoui‘ and Gourreau 2000; missing in Kovář 2007; syn. *Brumus quadripustulatus* used by Saharaoui et al. 2014)

*Parexochomus nigripennis* (Erichson, 1843) (syn. *Exochomus nigripennis* used by Saharaoui‘ and Gourreau 2000 and Saharaoui et al. 2014; Kovář 2007)

*Parexochomus pubescens* (Küster, 1848) (syn. *Exochomus pubescens* used by Saharaoui‘ and Gourreau 2000 and Saharaoui et al. 2014; Kovář 2007)

**Coccidulini**

*Rhyzobius chrysomeloides* (Herbst, 1793) (source: Saharaoui‘ and Gourreau 2000; Kovář 2007; Saharaoui et al. 2014)

*Rhyzobius litura* (Fabricius, 1787) (Kovář 2007)

*Rhyzobius lophantae* (Blaisdell, 1892) (source: Saharaoui‘ and Gourreau 2000; occurrence confirmed by Kherbouche et al. 2015; Kovář 2007; Saharaoui et al. 2014)

*Tetrabrachys cordicollis* (Guérin-Méneville, 1844) (Kovář 2007)

*Tetrabrachys cribratellus* (Fairmaire, 1876) (Kovář 2007)

*Tetrabrachys volkonskyi* (Peyerimhoff, 1943) (Kovář 2007)

**Coccinellini**

*Adalia bipunctata* (Linnaeus, 1758) (source: Saharaoui‘ and Gourreau 2000; missing in Kovář 2007; Saharaoui et al. 2014) *

*Adalia decempunctata* (Linnaeus, 1758) (not *A. decimpunctata* as misspelled by Saharaoui‘ and Gourreau 2000; Kovář 2007; Saharaoui et al. 2014) *

*Bulaea lividula* Mulsant, 1850 (Kovář 2007)

*Calvia quatuordecimguttata* (Linnaeus, 1758) (source: Saharaoui‘ and Gourreau 2000; missing in Kovář 2007; Saharaoui et al. 2014)

*Ceratomegilla notata* (Laicharting, 1781) (syn. *Semiadalia notata* used by Frah et al. 2009; missing in Kovář 2007)

*Ceratomegilla undecimnotata* (Schneider, 1792) (syn. Hippodamia (Semiadalia) undecimnotata used by Saharaoui‘ and Gourreau 2000; missing in Kovář 2007; Saharaoui et al. 2014)

*Cheilomenes propinqua* (Mulsant, 1850) (Kovář 2007)

*Coccinella septempunctata* Linnaeus, 1758 (syn. *C. algerica* Kovář 1977 used by Saharaoui‘ and Gourreau 2000; Kovář 2007; occurrence confirmed by Frah et al. 2009; Saharaoui et al. 2014) *

*Coccinella undecimpunctata* Linnaeus, 1758 (source: Saharaoui‘ and Gourreau 2000; Kovář 2007; Saharaoui et al. 2014)

*Harmonia axyridis* (Pallas, 1773) (**new record**) *

*Harmonia quadripunctata* (Pontoppidan, 1763) (Kovář 2007)

*Hippodamia tredecimpunctata* (Linnaeus, 1758) (source: Saharaoui‘ and Gourreau 2000; Kovář 2007; Saharaoui et al. 2014)

*Hippodamia variegata* (Goeze, 1777) (as H. (Adonia) variegata by Saharaoui‘ and Gourreau 2000; Kovář 2007; occurrence confirmed by Frah et al. 2009; Saharaoui et al. 2014) *

*Myrrha octodecimguttata* (not *M. octodecimpunctata* as misspelled by Saharaoui‘ and Gourreau 2000 and Saharaoui et al. 2014; Kovář 2007)

*Myrrha thuriferae* (Sicard, 1923) (Kovář 2007)

*Oenopia conglobata* (Linnaeus, 1758) (**new record**) *

*Oenopia doublieri* (Mulsant, 1846) (source: Saharaoui‘ and Gourreau 2000; Kovář 2007; Saharaoui et al. 2014) *

*Oenopia lyncea* (Olivier, 1808) (source: Saharaoui‘ and Gourreau 2000; Kovář 2007; Saharaoui et al. 2014)

*Propylea quatuordecimpunctata* (Linnaeus, 1758) (not *P. quatuordecimpuntata* as misspelled by Saharaoui‘ and Gourreau 2000; missing in Kovář 2007; Saharaoui et al. 2014)

*Psyllobora vigintiduopunctata* (Linnaeus, 1758) (source: Saharaoui‘ and Gourreau 2000; Kovář 2007; Saharaoui et al. 2014) *

*Tytthaspis phalerata* (Costa, 1849) (source: Saharaoui‘ and Gourreau 2000; Kovář 2007; Saharaoui et al. 2014)

**Epilachnini**

*Chnootriba elaterii* (Rossi, 1794) (syn. *Henosepilachna elaterii* used by Saharaoui‘ and Gourreau 2000; Kovář 2007; Saharaoui et al. 2014)

*Henosepilachna angusticollis* (Reiche, 1862) (Kovář 2007)

*Henosepilachna argus* (Geoffroy, 1785) (source: Saharaoui‘ and Gourreau 2000; Kovář 2007; Saharaoui et al. 2014)

**Hyperaspidini**

*Hyperaspis algirica* Crotch, 1874 (not *H. algerica* as misspelled by Saharaoui‘ and Gourreau 2000; Kovář 2007; Saharaoui et al. 2014)

*Hyperaspis duvergeri* Fürsch, 1985 (Kovář 2007) *

*Hyperaspis guttulata* Fairmaire, 1870 (Kovář 2007)

*Hyperaspis marmottani* (Fairmaire, 1868) (source: Saharaoui‘ and Gourreau 2000; Kovář 2007; Saharaoui et al. 2014) *

*Hyperaspis pseudopustulata* Mulsant, 1853 (Kovář 2007)

*Hyperaspis teinturieri* Mulsant & Godart, 1869 (Kovář 2007)

**Noviini**

*Novius cruentatus* Mulsant, 1846 (Kovář 2007)

*Rodolia cardinalis* (Mulsant, 1850) (source: Saharaoui‘ and Gourreau 2000; Kovář 2007; Saharaoui et al. 2014) *

**Platynaspidini**

*Platynaspis luteorubra* (Goeze, 1777) (source: Saharaoui‘ and Gourreau 2000; Kovář 2007; Saharaoui et al. 2014)

**Scymnini**

*Clitostethus arcuatus* (Rossi, 1794) (source: Saharaoui‘ and Gourreau 2000; missing in Kovář 2007; Saharaoui et al. 2014)

*Diomus rubidus* (Motschulsky, 1837) (Kovář 2007)

Nephus (Bipunctatus) bicinctus (Mulsant & Godart, 1870) (Kovář 2007)

Nephus (Bipunctatus) bipunctatus (Kugelann, 1794) (Saharaoui et al. 2014; missing in Kovář 2007)

Nephus (Bipunctatus) conjunctus (Wollaston, 1870) (Kovář 2007)

Nephus (Sidis) hiekei (Fürsch, 1965) (Kovář 2007)

Nephus (Sidis) levaillanti (Mulsant, 1850) (syn. *Scymnus levaillanti* used by Saharaoui‘ and Gourreau 2000; missing in Kovář 2007)

Nephus (Nephus) ludyi (Weise, 1879) (Kovář 2007)

Nephus (Bipunctatus) peyerimhoffi (Sicard, 1923) (source: Saharaoui‘ and Gourreau 2000; Kovář 2007; Saharaoui et al. 2014) *

Nephus (Nephus) quadrimaculatus (Herbst, 1783) (source: Saharaoui‘ and Gourreau 2000; missing in Kovář 2007; Saharaoui et al. 2014)

Nephus (Nephus) redtenbacheri (Mulsant, 1846) (Kovář 2007)

*Scymniscus splendidulus* (Stenius, 1952) (Kovář 2007)

Scymnus (Scymnus) apetzi Mulsant, 1846 (source: Saharaoui‘ and Gourreau 2000; missing in Kovář 2007; Saharaoui et al. 2014)

Scymnus (Scymnus) bivulnerus Baudi di Selve, 1894 (source: Saharaoui‘ and Gourreau 2000; Kovář 2007; Saharaoui et al. 2014)

Scymnus (Mimopullus) fulvicollis Mulsant, 1846 (syn. *Pullus fulvicollis* used by Saharaoui‘ and Gourreau 2000; Kovář 2007; Saharaoui et al. 2014)

Scymnus (Scymnus) interruptus (Goeze, 1777) (source: Saharaoui‘ and Gourreau 2000; Kovář 2007; Saharaoui et al. 2014)

Scymnus (Scymnus) laetificus Weise, 1879 (Kovář 2007)

Scymnus (Scymnus) marginalis (Rossi, 1794) (Kovář 2007)

Scymnus (Mimopullus) marinus (Mulsant, 1850) (syn. *Mimopullus mediterraneus* Iablokoff-Khnzorian, 1972 used by Saharaoui‘ and Gourreau 2000; Kovář 2007; Saharaoui et al. 2014)

Scymnus (Scymnus) nubilus (Mulsant, 1850) (Saharaoui et al. 2014; missing in Kovář 2007)

Scymnus (Scymnus) pavesii Canepari, 1983 (Kovář 2007)

Scymnus (Scymnus) rufipes (Fabricius, 1798) (source: Saharaoui‘ and Gourreau 2000; Kovář 2007; Saharaoui et al. 2014)

Scymnus (Pullus) subvillosus (Goeze, 1777) (syn *Pullus subvillosus* used by Saharaoui‘ and Gourreau 2000; Kovář 2007 Saharaoui et al. 2014)

Scymnus (Scymnus) suffrianioides Sahlberg, 1913 (syn. *S. pallipediformis* Günther, 1958 used by Saharaoui‘ and Gourreau 2000 and Saharaoui et al. 2014; missing in Kovář 2007) *

Scymnus (Pullus) suturalis Thunberg, 1795 (syn. *Pullus suturalis* used by Saharaoui‘ and Gourreau 2000; Kovář, 2007; Saharaoui et al. 2014)

**Stethorini**

*Stethorus pussilus* (Herbst, 1797) (syn. *S. punctillum* (Weise, 1891) used by Saharaoui‘ and Gourreau 2000 and Saharaoui et al. 2014; occurrence confirmed by Idder and Pintureau 2008; missing in Kovář 2007) *

**Sticholotidini**

*Coelopterus salinus* Mulsant & Rey, 1852 (Kovář 2007)

*Pharoscymnus numidicus* (Pic, 1900) (not *P. numidicus* as misspelled by Saharaoui‘ and Gourreau 2000; Kovář 2007; Saharaoui et al. 2014)

*Pharoscymnus ovoideus* Sicard, 1929 (source: Saharaoui‘ and Gourreau 2000; Kovář 2007; Saharaoui et al. 2014)

*Pharoscymnus setulosus* (Chevrolat, 1861) (source: Saharaoui‘ and Gourreau 2000; Kovář 2007; Saharaoui et al. 2014)

*Pharoscymnus sexguttatus* (Pic, 1926) (Kovář 2007)

## Discussion

*Chilocorus cacti* was introduced in Algeria but probably did not establish itself (Smirnoff 1957). *Coccinella algerica* Kovář, 1977 was described based on small morphological differences of North African populations originally thought to be *C. septempunctata*. Marin et al. (2010) demonstrated that these two species do not form genetically distinct lineages and synonymized *C. algerica* with *C. septempunctata*.

**Figure 1.**
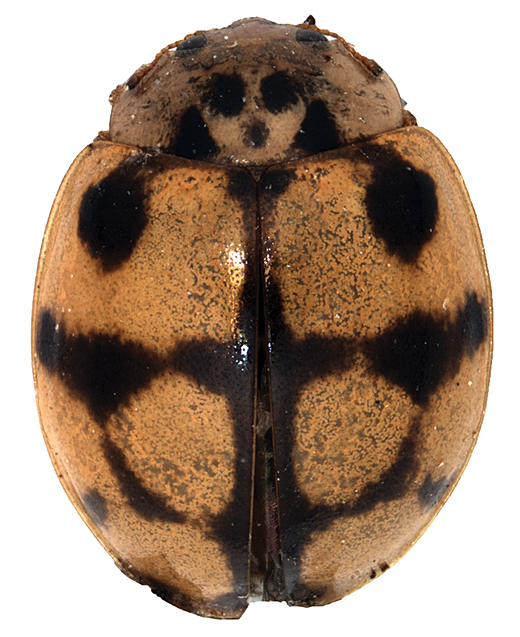
*Adalia decempunctata* found on *Ficus retusa* in El Harrach, Alger, 29 April 2017.

*Adalia decempunctata* was previously known from Algeria. Specimens of *Adalia decempunctata* found during our survey bear a mixture of characters of *A. decempunctata* and *A. conglomerata* (see Table 3 and Fig. 1). The former lives on a wide variety of woody plants, while *A. conglomerata* is a specialist on conifers, mainly spruce in Central Europe. Differences in the shape of male genitalia are generally small within *Adalia* to be used for clear species identification.

**Table 3.** Character states for *Adalia decempunctata*, *A. conglomerata*, and the specimens from El Harrach from 29 April 2017.

Character	*Adalia conglomerata*	*Adalia decempunctata*	Specimen 29/4/2017
Subapical elytral keel	absent	usually present	absent
Elytral background	yellow	variable	yellow
Shape of spots	deltoid	variable	deltoid
Length to width ratio	1.5	1.4	1.4
Tarsal claws	with tiny tooth	with large tooth	with large tooth
Body to scutellum ratio	25–30	15–18	25
Host plant	conifers	trees	*Ficus*

**Figure 2.**
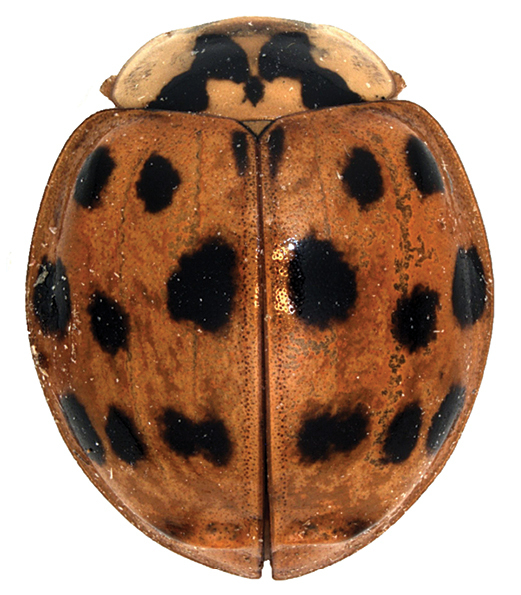
*Harmonia axyridis* found on *Prunus persica* (peach) in Mouzaia, Blida, 10 April 2017.

The occurrence of the invasive alien species *H. axyridis* in Algeria confirms predictions of its potential distribution made by Poutsma (2008) using a CLIMEX model. Although meanwhile it has been found in a few countries with wet tropical climate (Kenya: Nedvěd et al. 2011; Tanzania: Nedvěd and Háva 2016), and in dry tropical desert (Biranvand et al., in press) not predicted by the model, it probably did not establish itself there. The climate and host plants present in north Algeria and the occurrence of prey species and other predator ladybirds found during our study suggested establishment and future spread of *H. axyridis* in Algeria. The first specimen found was a male (Fig. 2), the second a female, both with well-developed elytral ridge, belonging to the form succinea. The establishment of the species was confirmed by occurrence of many larvae and pupae in 2018. All adults found in 2018 were of form succinea, which is the most common colour form in the native Chinese as well as in most invasive populations (Roy et al. 2016).

**Figure 3.**
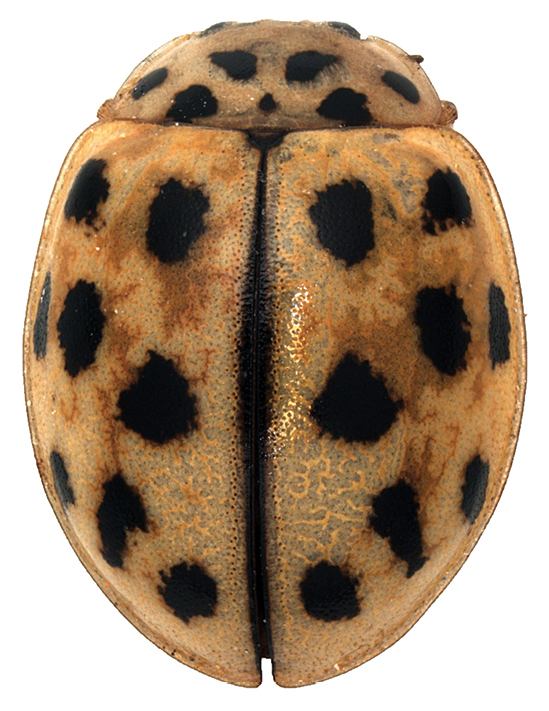
*Oenopia conglobata* found on *Quercus ilex* in El Harrach, 13 April 2017.

*Oenopia conglobata* is a common tree inhabiting predatory ladybird living in most European countries and as a subspecies in large parts of Asia. The specimen collected in Algeria has yellow elytral background (Fig. 3), while it is usually pink or beige in Europe. Additionally, the spots are rather small, while in many European individuals, at least some spots fuse together (Nedved 2015).
